# Assessing the Diagnostic Performance of Triglyceride–Glucose–Based Indices for Early Detection of Metabolic Syndrome in Paediatric Down Syndrome

**DOI:** 10.1111/jir.70134

**Published:** 2026-06-24

**Authors:** Valeria Calcaterra, Lucia Labati, Alessandra Anna Gazzarri, Alice De Lorenzo, Ilaria Anna Maria Scavone, Gianvincenzo Zuccotti

**Affiliations:** ^1^ Pediatric and Adolescent Unit, Department of Internal Medicine University of Pavia Pavia Italy; ^2^ Pediatric Department Buzzi Children's Hospital Milan Italy; ^3^ Department of Biomedical and Clinical Science “L. Sacco” University of Milan Milan Italy; ^4^ Associazione Vivi Down Onlus Milan Italy

**Keywords:** children, Down syndrome, HOMA‐IR, metabolic syndrome, paediatrics, triglyceride–glucose–based indices

## Abstract

**Background:**

Down syndrome (DS) youth have an increased risk of metabolic syndrome (MetS). This study assessed the diagnostic performance of the triglyceride–glucose index (TyG) and its derivatives—TyG‐BMI, TyG‐waist circumference (WC) and TyG‐waist‐to‐height ratio (WHtR) in detecting MetS compared with traditional markers.

**Methods:**

We retrospectively analysed data from 60 DS patients and 40 controls; IR was estimated using HOMA‐IR, TyG, TyG‐BMI, TyG‐WC and TyG‐WHtR indices. MetS was defined by the presence of at least three of the following criteria: BMI *z*‐score ≥ 2 SD and/or WC/H ratio ≥ 0.5; fasting glucose > 100 mg/dL and/or pathological HOMA‐IR; dyslipidaemia; hypertension.

**Results:**

MetS prevalence was 15% in DS. HOMA‐IR showed the highest sensitivity (0.667) and specificity (0.80) to detect MetS, while TyG demonstrated similar performance (sensitivity 0.667; specificity 0.843) and the best overall accuracy (0.817). Composite indices showed moderate accuracy but high specificity.

**Conclusions:**

TyG, being simple and cost‐effective, may be a valuable alternative for early MetS detection in DS.

- Main finding of your work
  ○ Children and adolescents with Down syndrome (DS) are at higher risk of metabolic syndrome (MetS), and the Triglyceride‐Glucose (TyG) index shows diagnostic accuracy comparable to HOMA‐IR in detecting it.
  ○ TyG demonstrated the highest overall accuracy (0.817), making it a reliable, practical alternative for identifying MetS in this population.
- The impact of it for people with intellectual disabilities and for the research community
  ○ The study enables early, low‐cost detection of cardiometabolic risk in individuals with intellectual disabilities, improving prevention and care.
  ○ It provides the research community with a simple, evidence‐based tool to advance studies on metabolic health in Down syndrome and similar conditions.

## 1 Introduction

Metabolic syndrome (MetS) is a cluster of metabolic and cardiovascular risk factors that significantly increase the likelihood of developing type 2 diabetes (T2D) and cardiovascular disease (CVD) (Neeland et al. 2024; Obeidat et al. 2025). In children and adolescents with Down syndrome (DS), the risk of MetS appears particularly high due to their increased predisposition to obesity, insulin resistance (IR), hypothyroidism and reduced physical activity (Molinari et al. 2024; Moreau et al. 2021; Hetman and Barg 2022).

The prevalence of MetS in the general paediatric population varies according to the diagnostic criteria applied, ranging from 3% to 5% in children and up to 17% in Italian adolescents, with substantially higher rates among those with obesity (Valerio et al. 2024; Zhang et al. 2025; Orsini et al. 2023). In children with DS, the prevalence of overweight and obesity has been reported to be up to twice that of their peers, accompanied by a higher frequency of dyslipidaemia and impaired glucose tolerance, suggesting an earlier and more pronounced risk of MetS (Molinari et al. 2024; Hetman and Barg 2022; Magge et al. 2019; Pecoraro et al. 2024).

IR is considered the central pathogenic mechanism of MetS (Thomaz et al. 2024; Zheng et al. 2025; Hamooya et al. 2025; Islam et al. 2024). Mechanistically, IR contributes to impaired glucose uptake, compensatory hyperinsulinaemia, altered lipid metabolism, endothelial dysfunction and increased cardiometabolic risk, thereby promoting the progression from early metabolic impairment to overt MetS, T2D and cardiovascular complications (Iafusco et al. 2023). In children with DS, several studies have demonstrated higher basal insulin levels and reduced insulin sensitivity, independent of adiposity (Molinari et al. 2024; Dierssen et al. 2020; Fonseca et al. 2005). Moreover, DS has been increasingly recognized as a condition associated with altered metabolic regulation and impaired insulin signalling, which may further contribute to peripheral and systemic metabolic dysfunction (Molinari et al. 2024; Moreau et al. 2021; Hetman and Barg 2022). This highlights the importance of early IR assessment in this population to prevent progression to T2D and cardiovascular complications.

The hyperinsulinaemic–euglycaemic clamp is the gold standard for assessing IR, but its application in paediatric clinical practice is limited by its invasiveness, costs and complexity (Tagi et al. 2022; Kim 2009). Therefore, simpler surrogate indices have been developed. Among them, the Homeostasis Model Assessment of Insulin Resistance (HOMA‐IR) is widely used, but it requires insulin measurement, which is not routinely available in all laboratories (Tagi et al. 2022a; Singh 2010; Kosmas et al. 2024; Esteghamati et al. 2010). A promising alternative is the triglyceride‐glucose index (TyG) and its anthropometric derivatives (TyG‐body mass index [BMI], TyG‐waist circumference [WC], TyG‐waist‐to‐height ratio [WHtR]) (Calcaterra et al. 2026; Aslan Çin et al. 2020; Dundar et al. 2023). These indices, based solely on fasting glucose and triglycerides, are simple, inexpensive and reproducible tools, already validated in both paediatric and adult populations as early markers of MetS, T2D and CVDs (Yang et al. 2024; Al Akl et al. 2023; Malek et al. 2021). However, their use in children with DS has been only marginally explored. This is particularly relevant because children and adolescents with DS are characterized by a distinctive metabolic profile and anthropometric phenotype.

Based on these considerations, the aim of our study was to evaluate the diagnostic performance of the TyG index and its anthropometric derivatives, in comparison with traditional markers, surrogate markers of insulin resistance, for identifying MetS in individuals with DS We seek to determine whether these indices can serve as simple and reliable tools for the early detection of MetS, thereby supporting timely prevention strategies for cardiovascular and metabolic complications in this vulnerable group.

## 2 Patients and Methods

### 2.1 Patients

We retrospectively analysed data from children and adolescents with genetically confirmed DS, who attended follow‐up evaluations at the Department of Paediatrics, Buzzi Children's Hospital (Milan, Italy), between March 2022 and May 2025.

Inclusion criteria were as follows: boys and girls aged ≤ 18 years at any Tanner stage (1 to 5) with confirmed diagnosis of DS, in euthyroid status and no ongoing pharmacological treatment known to affect glucose or lipid metabolism.

Exclusion criteria were as follows: presence of acute and/or chronic illnesses unrelated to DS; use of medications influencing metabolic parameters.

Forty healthy children and adolescents, matched for age and sex, who had been referred by their general practitioner or paediatrician for growth assessment, were enrolled as the control group.

Written informed consent was obtained from parents or legal guardians. The study protocol was approved by the Institutional Ethics Committee (Protocol number n. 2021/ST/207. Protocol register n. 0016834, 04/04/2022) and conducted in accordance with the Declaration of Helsinki and Good Clinical Practice guidelines.

### 2.2 Methods

#### 2.2.1 Clinical and Auxological Evaluation

All anthropometric parameters were collected following standardized protocols as previously reported (Calcaterra et al. 2026). Height was measured to the nearest 1 mm using a Harpenden stadiometer, with participants standing barefoot. Weight was recorded to the nearest 0.1 kg using a calibrated scale, with children wearing only underwear. BMI was calculated as weight divided by squared height (kg/m^2^) and expressed as *z*‐scores according to WHO reference charts (World Health Organnization (WHO), n.d.). WC was measured with a flexible tape at the midpoint between the lowest rib and the iliac crest, and the WHtR was subsequently calculated. Blood pressure (BP) was measured twice on the right arm after the child had been seated quietly for at least 5 min, using a sphygmomanometer with an appropriately sized cuff; the average of the two measurements was used in the analysis. Pubertal development was assessed according to Tanner staging during physical examination and subjects were classified as prepubertal (Stage 1), early pubertal (Stages 2 and 3) or late pubertal to adult stage (Stages 4 and 5) (Marshall and Tanner 1969, 1970).

#### 2.2.2 Biochemical Evaluation

Fasting morning blood samples were obtained after an overnight fast (≥ 8 h). Analyses included the following: fasting blood glucose (FBG), fasting insulin, glycated haemoglobin (HbA1c), total cholesterol (TC), high‐density lipoprotein cholesterol (HDL‐C), low‐density lipoprotein cholesterol (LDL‐C), triglycerides (TG), glutamate–oxaloacetate transaminase (GOT), glutamate–pyruvate transaminase (GPT) and gamma‐glutamyl transferase (GPT).

FBG, TC, HDL‐C, LDL‐C, TG, GOT, GPT and GGT were measured using automated clinical chemistry assays on the Alinity c system (Abbott Laboratories). HbA1c was measured using a direct enzymatic assay on the Alinity c system (Abbott Laboratories). Serum insulin concentrations were measured using a solid‐phase, two‐site chemiluminescent immunometric assay on an IMMULITE 2000 analyser. According to the manufacturer's specifications, the intra‐assay and total assay coefficients of variation for insulin ranged from 3.3% to 5.5% and from 4.1% to 7.3%, respectively. For the other biochemical parameters, intra‐assay and total assay coefficients of variation were within the analytical performance ranges reported by the manufacturers and were routinely monitored through the clinical laboratory quality‐control procedures.

The following indices were considered as surrogate markers of:

‐HOMA1‐IR = [fasting glucose (mg/dL) × fasting insulin (μU/mL)]/405.

Because fasting glucose was expressed in mg/dL, the denominator 405 was used; this corresponds to the conventional formula using glucose in mmol/L divided by 22.5 (Matthews et al. 1985), because glucose values in mg/dL are converted to mmol/L by dividing by 18.

HOMA‐IR was selected because it is a widely used, simple and convenient surrogate index for assessing insulin resistance. This is particularly relevant in paediatric populations, where direct methods such as the hyperinsulinaemic–euglycaemic clamp, although considered the gold standard, are invasive, time‐consuming, expensive and not feasible for routine clinical practice or large‐scale studies (Tagi et al. 2022). Moreover, HOMA‐IR allows comparison with previously published paediatric reference values, including the pubertal‐stage‐specific cut‐offs adopted in our analysis (d'Annunzio et al. 2009)

– TyG = ln [TG (mg/dL) × FBG (mg/dL)/2] (Tang et al. 2025)
– TyG derivatives: TyG‐BMI = TyG × BMI; TyG‐WC = TyG × WC (cm); TyG‐WHtR = TyG × WHtR (Tang et al. 2025)

TyG‐derived indices were calculated to integrate the biochemical information provided by TyG with anthropometric measures of overall and central adiposity. This approach has been previously proposed to improve cardiometabolic risk stratification by combining glucose–lipid metabolism with body fat distribution (Di Bonito et al. 2025; Calcaterra et al. 2026). However, because these indices have not been specifically validated in paediatric DS populations, their use in the present study should be considered exploratory.

#### 2.2.3 Definition of Metabolic Syndrome

As previously reported (Calcaterra et al. 2023), MetS was defined as the presence of at least three of the following criteria:

– BMI *z*‐score ≥ + 2 SD and/or WC/H ratio ≥ 0.5 and/or pathological WC (Maffeis et al. 2008);
– FBG ≥ 100 mg/dL and/or HOMA‐IR ≥ 2.5 in prepubertal, or ≥ 4 in pubertal/postpubertal subjects (corresponding to the 95th percentile, as reported by d'Annunzio et al. 2009);
– Systolic BP (SBP) ≥ 130 mmHg and/or diastolic BP (DBP) ≥ 85 mmHg (Zimmet et al. 2007);
– TG ≥ 100 mg/dL (age < 10 years) or ≥ 130 mg/dL (age ≥ 10 years) (Expert Panel on Detection, Evaluation, and Treatment of High Blood Cholesterol in Adults 2001).
– HDL‐C < 40 mg/dL in females or < 50 mg/dL in males (Zimmet et al. 2007).

To define MetS, we applied a tailored combination of existing criteria specifically selected for their relevance to paediatric populations and their suitability across age groups. The use of BMI *z*‐scores and WHtR provided a standardized, age‐independent measure of central adiposity. Pathological waist circumference was also included as an additional marker of abdominal adiposity, consistent with paediatric MetS classifications in which central obesity represents a key component (Wasniewska et al. 2023). Blood pressure and HDL‐C alteration were defined according to the IDF paediatric/adolescent MetS criteria (Zimmet et al. 2007). Gluco‐metabolic dysfunction was assessed through pathological FBG and/or IR levels, acknowledging that impaired fasting glucose is uncommon in childhood and that IR often precedes glucose abnormalities. Given the influence of pubertal transition on insulin dynamics, we used age‐specific thresholds for triglycerides and pubertal‐stage‐specific cut‐offs for HOMA‐IR to better capture physiological variability.

This approach aimed to enhance early detection of metabolic alterations and improve the accuracy of IR and MetS classification in children and adolescents.

### 2.3 Statistical Analysis

Continuous variables were expressed as mean ± standard deviation (SD) or, when not normally distributed, as median and interquartile range (IQR). Categorical variables were described as absolute numbers and percentages. Comparisons between groups for quantitative measures were carried out using one‐way analysis of variance (ANOVA) for independent samples, adopting a confidence level of 95%. Logistic regression models were applied to assess the significance of the area under the receiver operating characteristic (ROC) curve (AUC). A two‐tailed *p* value < 0.005 was considered statistically significant.

For each index (TyG, TyG‐BMI, TyG‐WC, TyG‐WHtR), distributions were divided into quartiles, with particular focus on the upper quartile (Q3). The third quartile was chosen as the threshold for further analyses because it represents a robust cut‐off that is less affected by outliers and extreme values than the mean, and it isolates the highest 25% of the sample, which is of particular interest in identifying children at increased metabolic risk. Unlike the median, which simply splits the dataset in two, Q3 is more informative in stratifying higher‐risk subgroups. In the absence of universally validated paediatric thresholds for TyG‐related indices, Q3 was considered a pragmatic and reproducible approach.

Within each subgroup, patients meeting criteria for MetS were identified, and for each marker, contingency tables (2 × 2 matrices) were created by comparing diagnostic test results with actual disease status. Sensitivity, specificity, positive predictive value (PPV) and negative predictive value (NPV) were calculated to evaluate diagnostic performance.

ROC curves were then constructed for each index and compared against conventional diagnostic parameters of MetS. Each curve was generated by plotting sensitivity versus 1‐specificity across the full range of cutoff values within the interquartile distribution. The AUC was computed using trapezoidal numerical integration, with values closer to 1.0 indicating superior diagnostic accuracy.

All statistical analyses were performed using R software (version 4.4, R Foundation for Statistical Computing, Vienna, Austria). To improve reliability, results were cross‐checked with calculations obtained in Microsoft Excel with advanced data analysis functions enabled.

## 3 Results

The study included 60 individuals with DS and 40 control subjects. The mean age did not differ significantly between groups (*p* = 0.989), and the sex distribution was comparable (DS: 32 males [M]/28 females [F]; controls: 26 M/14 F) (Table 1).

**TABLE 1:** Mean values of clinical and metabolic indices.

Features	Controls (*n* = 40)	Down syndrome (*n* = 60)	*p*
Age (years)	9.96 ± 2.29	9.97 ± 4.93	0.989
Sex (M/F)	M = 26 F = 14	M = 32 F = 28	0.303
Pubertal stage			
Stage 0	15	30	0.294
Stage 1	17	11	
Stage 2	8	19	
WC (cm)	63.16 ± 8.47	64.60 ± 19.45	0.615
Weight (kg)	38.19 ± 14.20	32.21 ± 17.64	0.065
Height (cm)	145.74 ± 17.74	122.35 ± 23.97	< 0.001
BMI (kg/m^2^)	17.28 ± 2.94	19.69 ± 5.53	0.006
BMI *z*‐score	−0.40 ± 1.29	0.48 ± 1.38	0.002
WHtR	0.43 ± 0.04	0.53 ± 0.10	< 0.001
Fasting blood glucose (mg/dL)	75.10 ± 8.07	90.38 ± 14.35	< 0.001
Insulin (U/mL)	5.86 ± 5.60	11.79 ± 11.25	< 0.001
HOMA‐IR	1.09 ± 0.99	2.80 ± 3.04	< 0.001
HDL cholesterol (mg/dL)	58.30 ± 7.31	62.29 ± 44.51	0.513
Total cholesterol (mg/dL)	139.13 ± 25.61	128.95 ± 41.80	0.139
Triglycerides (mg/dL)	60.28 ± 14.63	79.98 ± 22.73	< 0.001
GOT (U/L)	23.44 ± 5.46	39.81 ± 19.71	< 0.001
GPT (U/L)	13.08 ± 3.01	26.82 ± 16.67	< 0.001
GGT (U/L)	11.50 ± 1.58	14.64 ± 5.37	0.002
Systolic blood pressure (mmHg)	101.13 ± 7.88	98.05 ± 14.27	0.170
Diastolic blood pressure (mmHg)	65.23 ± 5.45	60.83 ± 9.71	0.005
TyG	7.69 ± 0.27	8.14 ± 1.83	< 0.001
TyG‐BMI	133.26 ± 31.18	159.93 ± 59.07	< 0.001
TyG‐WC	485.94 ± 69.32	526.32 ± 193.02	0.092
TyG‐WHtR	3.34 ± 0.35	4.29 ± 1.24	< 0.001

Abbreviations: BMI = body mass index; Homeostasis Model Assessment of Insulin Resistance = HOMA‐IR; TyG = triglyceride‐glucose index; WC = waist circumference; WHtR = waist‐to‐height ratio.

### 3.1 Anthropometric and Clinical Characteristics

Children with DS were significantly shorter than controls (*p* < 0.001) and exhibited higher BMI values (*p* = 0.006) and BMI *z*‐scores (*p* = 0.002). The WHtR was also elevated in the DS group (*p* < 0.001), indicating greater central adiposity (Table 1).

SBP did not differ significantly between groups (*p* = 0.170), while DBP was lower among DS subjects (*p* = 0.005). The results are summarized in Table 1.

### 3.2 Metabolic Parameters and Indices

As reported in Table 1, FBG levels were significantly higher in DS individuals compared with controls (*p* < 0.001), as were insulin concentrations (*p <* 0.001) and the HOMA‐IR (*p <* 0.001), suggesting increased IR among DS participants. Triglyceride levels were also higher in the DS group (*p <* 0.001), whereas total (*p* = 0.139) and HDL cholesterol showed no significant difference (*p =* 0.513). Serum hepatic enzymes were markedly elevated in the DS group: GOT (*p* < 0.001), GPT (*p* < 0.001) and GGT (*p* = 0.002).

MetS occurred in nine children with DS (15% of the total), without significant difference between sexes (*p* = 0.97). No cases were observed among controls.

All TyG‐related indices (TyG, TyG–BMI and TyG–WHtR) were significantly higher in the DS group compared with controls (*p* < 0.001), whereas TyG–WC did not reach statistical significance (*p* = 0.092). Quartile analysis confirmed a consistent upward shift of fasting glucose, HOMA‐IR, triglycerides, WHtR and TyG‐derived parameters among DS participants (Table 2).

**TABLE 2:** Quartile distribution of metabolic indices.

Variable	Quartile distribution	Controls	Down syndrome
WHtR	Min	0.36	0.31
	Q1	0.41	0.48
	Med	0.42	0.52
	Q3	0.46	0.56
	Max	0.59	0.78
Blood glucose	Min	59.00	62.00
	Q1	68.00	81.75
	Med	74.50	93.00
	Q3	80.00	102.00
	Max	90.00	114.00
HOMA‐IR	Min	0.09	0.05
	Q1	0.43	1.16
	Med	0.84	1.94
	Q3	1.39	2.77
	Max	5.71	16.83
HDL Cholesterol	Min	48.00	22.00
	Q1	51.00	37.75
	Med	55.50	45.00
	Q3	65.00	58.00
	Max	77.00	180.00
Triglycerides	Min	35.00	33.00
	Q1	51.00	67.00
	Med	60.50	76.00
	Q3	73.00	91.00
	Max	86.00	130.00
Systolic blood pressure	Min	90.00	60.00
	Q1	95.00	90.00
	Med	100.00	95.00
	Q3	105.00	110.00
	Max	120.00	130.00
Diastolic blood pressure	Min	55.00	35.00
	Q1	60.00	60.00
	Med	65.00	60.00
	Q3	70.00	66.25
	Max	80.00	80.00
TyG	Min	6.94	7.08
	Q1	7.55	7.91
	Med	7.77	8.18
	Q3	7.87	8.35
	Max	8.07	8.83
TyG BMI	Min	93.13	75.97
	Q1	115.66	134.02
	Med	127.38	149.63
	Q3	155.17	182.11
	Max	172.42	376.44
TyG WC	Min	353.48	294.08
	Q1	427.91	422.74
	Med	506.27	464.89
	Q3	543.15	597.44
	Max	599.60	934.72
TyG WHtR	Min	2.76	2.65
	Q1	3.08	3.79
	Med	3.33	4.28
	Q3	3.58	4.64
	Max	4.34	6.37

Abbreviations: BMI = body mass index; Homeostasis Model Assessment of Insulin Resistance = HOMA‐IR; Max = maximum; Med = median; Min = minumun; Q = quartile; TyG = triglyceride‐glucose index; WC = waist circumference; WHtR = waist‐to‐height ratio.

### 3.3 Diagnostic Performance

Specificity and sensitivity analyses (Table 3) revealed that HOMA‐IR demonstrated the highest sensitivity (0.667) and specificity (0.804) to detect MetS. Among the TyG‐derived indices, TyG showed comparable diagnostic performance (sensitivity = 0.667; specificity = 0.843). The composite indices TyG–BMI, TyG–WC and TyG–WHtR displayed moderate diagnostic accuracy, with consistently high specificity values ranging from 0.72 to 0.78; although their sensitivity values were generally lower (0.111–0.333).

**TABLE 3:** Specificity and sensitivity of metabolic indices.

Specificity						
Parameters	Controls			Down syndrome		
	Specificity	95% CI		Specificity	95% CI	
Glucose	1.000	1.000	1.000	0.863	0.776	0.950
BMI *z*‐score	0.975	0.868	0.999	0.882	0.800	0.964
HOMA‐IR	0.925	0.796	0.984	0.804	0.704	0.904
HDL cholesterol	0.975	0.868	0.999	0.608	0.484	0.732
Triglycerides	1.000	1.000	1.000	0.961	0.912	1.010
Systolic blood pressure	1.000	1.000	1.000	0.980	0.945	1.015
Diastolic blood pressure	1.000	1.000	1.000	1.000	1.000	1.000
Waist‐to‐height ratio	0.975	0.868	0.999	0.412	0.287	0.537
TyG	0.725	0.587	0.863	0.843	0.751	0.935
TyG‐BMI	0.750	0.616	0.884	0.765	0.658	0.872
TyG‐WC	0.750	0.616	0.884	0.725	0.612	0.838
TyG‐WHtR	0.750	0.616	0.884	0.784	0.680	0.888

Sensitivity						
	Controls			Down syndrome		
	Sensitivity	95% CI		Sensitivity	95% CI	
Glucose	—	—	—	1.000	1.000	1.000
BMI *z*‐score	—	—	—	0.111	0.032	0.190
HOMA‐IR	—	—	—	0.667	0.548	0.786
HDL cholesterol	—	—	—	0.889	0.810	0.968
Triglycerides	—	—	—	0.444	0.318	0.570
Systolic blood pressure	—	—	—	0.000	0.000	0.000
Diastolic blood pressure	—	—	—	0.000	0.000	0.000
Waist‐to‐height ratio	—	—	—	0.667	0.548	0.786
TyG	—	—	—	0.667	0.548	0.786
TyG‐BMI	—	—	—	0.222	0.117	0.327
TyG‐WC	—	—	—	0.111	0.032	0.190
TyG‐WHtR	—	—	—	0.333	0.214	0.452

Abbreviations: BMI = body mass index; Homeostasis Model Assessment of Insulin Resistance = HOMA‐IR; TyG = triglyceride‐glucose index; WC = waist circumference.

TyG index demonstrated higher overall diagnostic accuracy (0.817) compared with HOMA‐IR (0.783), with a similar NPV (HOMA‐IR 0.932 vs. TyG 0.935) and slightly improved PPV (HOMA‐IR 0.375 vs. TyG 0.429). Among the composite indices, TyG–BMI, TyG–WC and TyG–WHtR showed moderate levels of accuracy (0.683, 0.633 and 0.717, respectively). All of these indices were characterized by consistently high NPVs (> 0.82), but their PPVs remained limited (ranging from 0.067 to 0.214) (Table 4).

**TABLE 4:** Predictive positive value (PPV), negative predictive value (NPV) and discriminant capacity expressed as the area under the curve (AUC) of metabolic parameters.

Parameters	Controls							Down syndrome						
	PPV	95% CI	NPV	95% CI		AUC		PPV	95% CI		NPV	95% CI		AUC
Fasting blood glucose	0.000	0.000	0.000	1.000	1.000	1.000	1.000	0.563	0.437	0.689	1.000	1.000	1.000	0.883
BMI *z*‐score	0.000	0.000	0.000	1.000	1.000	1.000	0.975	0.143	0.054	0.232	0.849	0.758	0.940	0.767
HOMA‐IR	0.000	0.000	0.000	1.000	1.000	1.000	0.925	0.375	0.253	0.498	0.932	0.868	0.996	0.783
HDL cholesterol	0.000	0.000	0.000	1.000	1.000	1.000	0.975	0.286	0.172	0.400	0.969	0.925	1.000	0.650
Triglycerides	0.000	0.000	0.000	1.000	1.000	1.000	1.000	0.667	0.548	0.786	0.907	0.834	0.980	0.883
SBP	0.000	0.000	0.000	1.000	1.000	1.000	1.000	0.000	0.000	0.000	0.847	0.756	0.938	0.833
DBP	0.000	0.000	0.000	1.000	1.000	1.000	1.000	—	—	—	0.850	0.760	0.940	0.850
WHtR	0.000	0.000	0.000	1.000	1.000	1.000	0.975	0.167	0.073	0.261	0.875	0.791	0.959	0.450
TyG	0.000	0.000	0.000	1.000	1.000	1.000	0.725	0.429	0.304	0.554	0.935	0.873	0.997	0.817
TyG‐BMI	0.000	0.000	0.000	1.000	1.000	1.000	0.750	0.143	0.054	0.232	0.848	0.757	0.939	0.683
TyG‐WC	0.000	0.000	0.000	1.000	1.000	1.000	0.750	0.067	0.004	0.130	0.822	0.725	0.919	0.633
TyG‐WHtR	0.000	0.000	0.000	1.000	1.000	1.000	0.750	0.214	0.110	0.318	0.870	0.785	0.955	0.717

Abbreviations: AUC = area under curve; BMI = body mass index; HOMA‐IR = Homeostasis Model Assessment of Insulin Resistance; NPV = negative predictive value; PPV = positive predictive value; TyG = triglyceride–glucose index; WC = waist circumference; WHtR = waist‐to‐height ratio.

The discriminant capacity expressed as the area under the curve (AUC) varied among the evaluated indices. In individuals with DS, HOMA‐IR demonstrated a good ability to discriminate metabolic alterations, with an AUC of 0.735 (95% CI: 0.538–0.932). The TyG index exhibited a slightly higher AUC value of 0.755 (95% CI: 0.562–0.948), indicating comparable or marginally superior diagnostic performance to HOMA‐IR in identifying insulin resistance–related metabolic disturbances. Among the TyG‐derived indices, TyG–WHtR showed a modest discriminative ability (AUC = 0.559; 95% CI: 0.349–0.769), while TyG–BMI (AUC = 0.493; 95% CI: 0.288–0.699) and TyG–WC (AUC = 0.418; 95% CI: 0.223–0.613) demonstrated limited discriminatory power.

## 4 Discussion

Collectively, children with DS exhibited a distinctive anthropometric and metabolic profile characterized by reduced stature, increased adiposity, IR, hypertriglyceridaemia and elevated liver enzyme levels, leading to a higher prevalence of MetS. Overall, similarly to HOMA‐IR, the TyG index emerged as an accurate surrogate marker of IR, displaying good discriminative capacity and high NPV, which highlight its reliability for ruling out, rather than confirming, metabolic abnormalities. The derived indices (TyG–BMI, TyG–WC and TyG–WHtR) demonstrated only moderate diagnostic accuracy and did not improve predictive performance compared with the original TyG index. TyG index could provide an additional advantage in identifying children and adolescents with DS who are at higher risk of MetS. Although TyG‐derived indices offered limited predictive gain, they still provided complementary insights into the relationship between adiposity and metabolic impairment.

DS is the most common chromosomal disorder (Rafii et al. 2019) and is frequently associated with a complex metabolic phenotype characterized by excess body fat, reduced physical activity, altered energy balance, altered hormonal sensitivity and thyroid dysfunction (Moreau et al. 2021; Pecoraro et al. 2024; Fonseca et al. 2005; Calcaterra et al. 2023; Segura‐Jiménez et al. 2016; Binsaddiq et al. 2025; Giuriato et al. 2025), factors that collectively contribute to an elevated cardiometabolic risk compared with the general paediatric population. Previous studies have reported that approximately 12%–20% of children with DS meet the diagnostic criteria for MetS, showing early manifestations of IR, dyslipidaemia and hepatic dysfunction even before puberty (Moreau et al. 2021; Calcaterra et al. 2023; Rafii et al. 2019). Our findings confirm a significantly higher prevalence of MetS, by 15%, in the study group compared with control children. This evidence underscores the importance of implementing more effective strategies for the early identification of metabolic alterations in this paediatric population.

Even though the pathophysiological background of MetS is multifactorial, IR is widely recognized as a central mechanism driving metabolic dysfunction and the development of cardiovascular complications. (Magge et al. 2019; Fonseca et al. 2005; Zhao et al. 2023). IR contributes to impaired glucose uptake, compensatory hyperinsulinaemia and dysregulation of lipid metabolism, which together promote a proinflammatory and proatherogenic state. Moreover, IR is closely linked to visceral adiposity and altered adipokine secretion, further exacerbating endothelial dysfunction and increasing cardiovascular risk (Zhao et al. 2023; Cresswell et al. 2024; Chait and Den Hartigh 2020). Therefore, the early identification of subclinical IR is essential to prevent the development of MetS, type 2 diabetes and nonalcoholic fatty liver disease (Gesteiro et al. 2021). However, early screening alone is not sufficient to improve long‐term outcomes unless it is followed by targeted and feasible interventions. In children and adolescents with DS, preventive strategies should include individualized nutritional counselling, promotion of regular physical activity adapted to motor abilities and comorbidities (Rein et al. 2026; Giuriato et al. 2025), and close multidisciplinary follow‐up involving paediatricians, endocrinologists, dietitians and rehabilitation specialists. Future studies are needed to evaluate whether early identification of IR through simple indices such as TyG can effectively guide personalized interventions and improve cardiometabolic outcomes in this population.

In our cohort, fasting glucose, insulin and HOMA‐IR were markedly increased in DS individuals, confirming a higher degree of IR in this population (Fonseca et al. 2005). The HOMA‐IR index is widely recognized as a reliable surrogate marker for estimating insulin sensitivity, given its simplicity, good accuracy and well‐documented correlation with the euglycaemic–hyperinsulinaemic clamp, which remains the gold standard for assessing IR (So et al. 2020; Minh et al. 2021). However, despite its broad use, HOMA‐IR has several limitations. It requires insulin measurement, which may vary depending on the analytical method and is not always available in routine laboratory settings. Moreover, its reliability may decrease in individuals with impaired β‐cell function or altered hepatic insulin metabolism (Manley et al. 2008; Samavarchitehrani et al. 2025).

In this context, the TyG index has emerged as a practical, inexpensive and reproducible alternative, as it does not require insulin assessment. Several studies have demonstrated a strong correlation between TyG and HOMA‐IR, suggesting that TyG could serve as a useful surrogate indicator of IR, particularly in large‐scale or resource‐limited settings where insulin measurement is not feasible (Calcaterra et al. 2026; Dundar et al. 2023).

In our study, TyG values were significantly higher in DS participants than in controls, and the index demonstrated a good discriminative ability to detect MetS with an AUC of 0.755 (95% CI: 0.562–0.948), slightly outperforming HOMA‐IR (AUC = 0.735; 95% CI: 0.538–0.932). Moreover, TyG achieved sensitivity = 0.667, specificity = 0.843 and overall accuracy = 0.817, confirming its potential as a screening tool for early metabolic impairment, as previously reported in individuals with obesity (Kalyoncu and Kavrak Kursun 2025). The comparable or even superior performance of TyG relative to HOMA‐IR, combined with its ease of use and lower analytical cost, supports its application in paediatric DS settings, where frequent venipuncture and insulin assays can be difficult to perform. Therefore, while HOMA‐IR remains a valid and widely used tool, integrating alternative indices such as TyG may enhance the assessment of insulin resistance by combining accuracy, economic sustainability and greater accessibility.

By contrast, the TyG‐derived indices, which combine biochemical and anthropometric data, showed lower diagnostic accuracy, with AUC values ranging from 0.418 to 0.559 and overall accuracy between 0.63 and 0.72. Although these indices retained high specificity (0.72–0.78) and strong NPV (> 0.82), their low sensitivity (0.11–0.33) suggests limited added value over the standard TyG index in this population. In DS, the atypical body composition, characterized by reduced lean mass, altered fat distribution and differences in visceral‐to‐subcutaneous fat ratio (Binsaddiq et al. 2025; Pecoraro et al. 2023), may weaken the association between anthropometric and metabolic indices, thereby reducing the discriminative power of TyG‐related combinations.

Nevertheless, TyG‐derived indices still provide complementary information on how body‐fat distribution interacts with glucose–lipid homeostasis and IR (Tang et al. 2025; He et al. 2025). Their high specificity may help identify children with DS who are metabolically healthy despite increased BMI, supporting a more individualized approach to risk stratification.

Several limitations must be considered when interpreting these results. First, the retrospective design of the study limits the ability to establish causal relationships between TyG indices and the development of metabolic alterations. Longitudinal studies are needed to evaluate whether elevated TyG values can predict future onset of MetS or T2DM in DS populations. Second, the sample size was relatively small, particularly when stratifying by sex and pubertal stage, which may have reduced statistical power. Third, although pubertal‐stage‐specific HOMA‐IR thresholds were used, insulin resistance during puberty may be physiologically transient. Thus, some adolescents undergoing normal pubertal development may have been classified as insulin resistant. Moreover, due to the cross‐sectional nature of the study, we were unable to determine whether insulin resistance was transient or persistent over time.

Additionally, lifestyle and environmental factors, including dietary intake, physical activity and family history, were not controlled and their contribution to metabolic outcomes could not be fully assessed. In particular, dietary patterns and socioeconomic factors were not available in our dataset, although they may significantly influence obesity, IR and cardiometabolic risk in children and adolescents with DS. The absence of these variables may have limited our ability to fully account for potential confounding factors.

Another limitation concerns the definition of MetS components. In paediatric age, MetS diagnosis remains debated because no universally accepted classification is available, and no specific validated criteria exist for subjects with DS, despite their distinctive auxological profile, body composition and fat distribution. Therefore, we selected parameters commonly included in paediatric MetS classifications and adapted them to this population. BMI *z*‐score was used as a standardized indicator of overall adiposity adjusted for age and sex, whereas WHtR and pathological WC were included as complementary markers of central adiposity and cardiometabolic risk. Although WHtR is simple, relatively age‐ and sex‐independent and useful when BMI alone may not fully reflect body fat distribution, these measures assess different aspects of adiposity and may have introduced heterogeneity, with possible misclassification or overestimation of MetS prevalence. Moreover, the use of fixed blood pressure thresholds, rather than age‐, sex‐ and height‐specific paediatric percentiles, may have reduced diagnostic accuracy, particularly in younger children.

Finally, the lack of standardized paediatric or DS‐specific cut‐off values for TyG and related indices limits the generalizability of our findings and underscores the need for multicentric and multi‐ethnic studies to establish appropriate reference ranges.

## 5 Conclusions

Our findings confirm that the TyG index performs comparably to HOMA‐IR in identifying MetS among children with DS, while offering notable practical and economic advantages. Its exclusive reliance on glucose and triglyceride measurements, routinely available in clinical practice, makes it particularly suitable for longitudinal monitoring in this population. Although TyG‐derived indices did not enhance predictive accuracy in our cohort, they remain valuable complementary markers reflecting the complex relationship between adiposity and metabolic dysfunction. Incorporating TyG‐based screening into routine follow‐up programs for DS could support the early detection of metabolic alterations, enable timely preventive interventions and ultimately help reduce long‐term cardiometabolic risk in this vulnerable population.

## Funding

The authors have nothing to report.

## Ethics Statement

The study protocol received approval from the Institutional Review Board (Protocol number n. 2021/ST/207. Protocol register n. 0016834, 04/04/2022, ce Area 1 Milan, Italy).

## Consent

Upon receiving information regarding the study's nature, written informed consent was obtained from the parents/authorized caregivers of the participating children.

## Conflicts of Interest

The authors declare no conflicts of interest.

## Data Availability

Data are available on request from the authors.
